# The Global Registry of Biodiversity Repositories: A Call for Community Curation

**DOI:** 10.3897/BDJ.4.e10293

**Published:** 2016-08-26

**Authors:** David E. Schindel, Scott E. Miller, Michael G. Trizna, Eileen Graham, Adele E. Crane

**Affiliations:** ‡ Consortium for the Barcode of Life and Scientific Collection International, National Museum of Natural History, Smithsonian Institution, Washington, DC, United States of America; § Office of the Provost, Smithsonian Institution, Washington, DC, United States of America; | Consortium for the Barcode of Life, National Museum of Natural History, Smithsonian Institution, Washington, DC, United States of America; ¶ Scientific Collections International, National Museum of Natural History, Smithsonian Institution, Washington, DC, United States of America

**Keywords:** Collections, Repositories, Registry, institutionCode, collectionCode, Darwin Core Triplet, Authority File

## Abstract

The Global Registry of Biodiversity Repositories is an online metadata resource for biodiversity collections, the institutions that contain them, and associated staff members. The registry provides contact and address information, characteristics of the institutions and collections using controlled vocabularies and free-text descripitons, links to related websites, unique identifiers for each institution and collection record, text fields for loan and use policies, and a variety of other descriptors. Each institution record includes an institutionCode that must be unique, and each collection record must have a collectionCode that is unique within that institution. The registry is populated with records imported from the largest similar registries and more can be harmonized and added. Doing so will require community input and curation and would produce a truly comprehensive and unifying information resource.

## Introduction

Taxonomy and the monographs and journals that publish them (like this one) are founded on the documentation of specimens, their traits, and their distribution in time and space. The same is true for biogeographic and ecological studies such as surveys and inventories (e.g., Telfer et al. 2015). We call them “vouchers” because they vouch for the authenticity of raw data. Specimen-based biodiversity research is repeatable and its hypotheses are confirmable or refutable only because specimens can be found, re-examined, and analyzed using new techniques. The reproducibility of scientific findings has recently emerged as a critical issue for science policy (Yaffe 2015) and it applies to observational fields like taxonomy as well as experimental disciplines like chemistry. (Arnett 1970a, Arnett 1970b), and more recently (Chavan and Penev 2011), have argued that the raw data taken from voucher specimens are sufficiently important, in and of themselves, to merit publication, respectively, in “data documents” and “data papers”, separate from their subsequent analyses and interpretation in scholarly articles. In taxonomy, these data are taxonomic identifications, traits, occurrence locations and dates, and images, all associated with unique specimen identifiers. As collections specialists focus on digitizing specimens, we may be at risk of omitting critical pieces of information about voucher specimens cited in the literature, such as the location of the specimens and information on how to access them.

Our goal for the activity described here is to connect physical specimens to their citations in the literature as well as to their digitized records in collections databases and aggregators. The Global Registry for Biodiversity Repositories (GRBio) is an online resource hosted by the Smithsonian Institution in Washington, DC. Its mission is to connect published specimen references to physical specimens by making basic information about collections available. Where is it located? What are their policies concerning access and specimen loans? To whom should I address a loan request?

GRBio is an information resource whose success depends on the active participation of the community. This editorial calls on the taxonomic community for three things. First, we call on all repositories of biological reference specimens to contribute data to GRBio about their institutions and collections. Second, we ask for their involvement in maintaining data quality to keep the data current, reliable and unambiguous. Third, we call on the community to use GRBio as an authoritative information source that will make taxonomic research more open, unambiguous, and reproducible. As described below, ZooKeys and Pensoft Publishing have been the first to take this important step.

## What is GRBio?

GRBio (www.grbio.org) is an online Drupal-based database and portal that provides essential information about institutions that maintain collections, the biological collections they contain, and the people associated with those institutions and collections. This information includes: standard codes for institutions and collections; mailing and physical addresses; websites and links to online catalogs and web services; points of contact; characterizations of institutions and collections using controlled vocabularies; free-text descriptions; access, loan and use policies; and persistent identifiers for each record institution and collection record. Each institutional and collection record has an LSID (e.g., “urn:lsid:biocol.org:col:15670”, assigned by the Biodiversity Collections Index; see below) and/or a CoolURI assigned by GRBio (e.g., http://grbio.org/cool/v1fg-sphq). The portal provides basic and advanced search capabilities, as well as opportunities to download specific records or all records of institutions, collections, or staff members.

GRBio was developed by the Consortium for the Barcode of Life (CBOL) as an authoritative reference source for use in linking voucher specimens with DNA barcode records in GenBank. GenBank uses the “Darwin Core Triplet” (DwCT) as the standard format for identifying the voucher specimen from which a DNA sequence was obtained. The DwCT is a commonly used specimen identifier that combines three Darwin Core terms delimited by a colon (":"). The resulting format is institutionCode:collectionCode:catalogNumber. To be effective as a source of authoritative information, institutionCodes should be unique and unambiguous across all institutions. collectionCodes only need to be unique within an institution.

Guralnick et al. (2014) described a number of challenges in the use of DwCT as a unique specimen identifier. They stressed the need for better data curation before institutionCodes and collectionCodes could be used on a large scale. We propose GRBio as the platform for that data curation.

With the same goal of creating a single, comprehensive source of institutionCodes and collectionCodes, four other registries agreed to contribute their data into a merged database served through the GRBio portal. The datafields in the four registries overlapped to a significant degree but there were some differences in their structures and functions. The registries that contributed data to GRBio are:

- NCBI's Institution table and Collection table, compiled from the specimen information submitted with GenBank records;
- Biorepositories.org, CBOL's first attempt to construct a registry. It included records for institutions, collections within those institutions, and privately owned collections that were assigned to a virtual institution with the institutionCode “personal”;
- Index Herbariorum (IH), the well-established registry of herbaria that is hosted by the New York Botanical Garden. IH contains herbarium-level records and staff member records that include datafields unique to IH (e.g., year the herbarium was founded, geographic and taxonomic emphases, size of collection); and
- Biodiversity Collections Index (BCI), created by Roger Hyam and hosted by the Royal Botanic Garden Edinburgh for several years. BCI was taken off line after the launch of GRBio. BCI did not employ the collections-within-institutions structure. Rather, it treated all organizations as 'collections' that sometimes had a parent-child relationship to other collections. BCI assigned a Life Science Identifier (LSID) to each collection record. The records in BCI were imported principally from IH and the Insect and Spider Collections of the World, created by Arnett 1983, now curated as an online resource by Neal Evenhuis and hosted by the Bernice P. Bishop Museum.

The managers of these four registries consulted for a year and developed a consensus version that combined into one platform the critical elements of each. GRBio includes four content types:

- Institutions, 98+% of which have a unique institutionCode that refers unambiguously to one organization;
- Institutional or project collections, each associated with one institution. As used here, “institutional” collections have been formally accessioned and each has a system of catalogNumbers issued by that institution. “Project” collections belong to an institution but haven't yet been accessioned. They are generally overseen by researchers, not collection managers, and are identified by field numbers or other identifiers minted by the collector, rather than catalogNumbers issued by the institution (see IWGSC 2013);
- Personal collections that are privately owned and are associated with the virtual institutionCode “personal”; and
- Staff members.

Users can enter new records and update existing records through the online portal. No log-in is required. New and revised data records are not made public until they have been reviewed and approved by a moderator. Records typically remain in a non-public queue for several days until they have been checked and published.

GRBio was released by CBOL in June 2013 and its management was transferred to Scientific Collections International (SciColl) in December 2013. SciColl developed under the auspices of the OECD Global Science Forum (formerly the Megascience Forum) and is hosted by the Smithsonian Institution. SciColl promotes interdisciplinary research that relies on scientific collections in all disciplines, including, but not limited to, biodiversity. With the goal of increasing access to collections in other disciplines, SciColl began developing the Global Registry of Scientific Collections (GRSciColl) as a second portal that serves data to and from the same database as GRBio. GRSciColl was released in October 2014 and began accepting data on collections in anthropology, archaeology, biomedicine, earth and space sciences, and applied fields such as agriculture, technology and veterinary medicine. A third portal, U.S. Federal Scientific Collections (USFSC) displays data from the same database and stores approved data to it. USFSC encompasses scientific collections owned by the departments and agencies of the U.S. Federal government. It was developed by the Interagency Working Group on Scientific Collections, an activity of the White House National Science and Technology Council. The USFSC portal was created to make information about federal collections more accessible, in response to two Policy Memorandums from the White House Office of Science and Technology Policy (OSTP 2010 and OSTP 2014) and Section 104 of the America COMPETES Reauthorization Act of 2010.

With support from the U.S. National Science Foundation, CBOL and SciColl held workshops in April 2015 and February 2016 to gather community feedback on GRBio and GRSciColl, respectively. SciColl has begun to implement the advice provided during these workshops as first steps in the long-term improvement of the portals and their underlying database. Future improvements include systems for synchronizing separate registries of collections and setting up web service APIs.

## ﻿﻿﻿Resolving Am﻿biguities﻿﻿﻿﻿

Authors of taxonomic articles and monographs have traditionally referred to specimens using two elements: (1) the abbreviation, code or acronym associated with the institution in which the specimen is stored, and (2) the catalog number on its label. The first element can be thought of as a kind of institutional trademark, instantly recognizable to most community members (e.g., USNM, AMNH, MCZ). Unfortunately there has never been an authoritative list for these trademarks or a protocol for regulating them. Many of them, in fact, are neither unique nor unambiguous. The same trademark can be used for different institutions in botany, zoology and microbiology. Institutions in different countries (and occasionally in the same country) sometimes use the same trademark. To complicate this name-space even further, some institutions have changed their trademarks over time due to “rebranding” initiatives, takeovers or mergers.

When the four datasets were merged into a single database there were approximately 7,000 institution records. Of these, ~10% had an institutionCode that was associated with more than one institution. Of the 299 ambiguous institutionCodes at the time, 200 involved “collisions” between herbaria in IH and museums or other organizations imported from other registries. IH agreed to the addition of the suffix "<IH>" to the institutionCodes of their records, thereby reducing the number of ambiguous institutionCodes to approximately 100. Some multiple uses may result from separate records for the same institution with a slightly different institutional name. Others are genuine ambiguities involving separate institutions. Some of the remaining ambiguities are noteworthy; five institutions use the institutionCode "SM". Some institutions have changed their institutionCodes in the past to resolve an ambiguity but the change had not been entered into GRBio. When the new institutionCode is entered, the outdated ones are maintained in the database the Status datafield set to “Inactive”. An annotation is added to the Description field with a pointer to the current record for that institution (with the newer unique and unambiguous institutionCode). This ensures that references in the literature that use older codes are still resolvable and can be traced to the institution through updated codes. When new institutions are created in GRBio or existing records are updated, each proposed institutionCodes is checked to ensure that the database has no other “Active” records with that code. Records with ambiguous institutionCodes cannot be added to the database.

Participants in the NSF workshop mentioned above recommended synchronizing GRBio with the contents of other registries of biodiversity collections that have not yet been integrated into GRBio. Doing so would make GRBio more comprehensive and useful, but without extra measures it would add more cases of multiple-use, ambiguous institutionCodes. Some of these ambiguities stem from mixing codes for institutions, collections within institutions, and collectors in the same data table. For example, The Insect and Spider Collections of the World contains more than 1,800 “codens”, each comprising three to six letters. The listing for each coden includes either:

- The name of an institution that holds a collection of insects and/or spiders (e.g., SUI: University of Iowa, Museum of Natural History). SUI has the same meaning in GRBio;
- The name of a collection of insects and/or spiders (e.g., UAM: University of Alaska Museum, Entomology Collection). In GRBio, UAM is the institutionCode for the museum and "Ento" is the collectionCode for its insect collection;
- A pointer to another coden, presumably the one currently used by the institution (332 cases). These are equivalent to institutionCode with "Inactive" status in GRBio; or
- A coden for an individual (388 cases), equivalent to Personal Collections in GRBio.

The American Society of Mammologists periodically publishes updates to "Mammal Collections of the Western Hemisphere" (Hafner et al. 1997). The American Society of Ichthyologists and Herpetologists publishes a similar "Standard Symbolic Codes for Institutional Resource Collections in Herpetology & Ichthyology" (Sabaj 2016). These compilations also contains a mixture of institutions and collections (in the sense used by GRBio), each assigned an identification code.

collectionCodes are also critical for ensuring that references to published specimens are unambiguous. Any particular catalogNumber could be used (and usually has been used) in more than one departmental collection in an institution (plants, insects, fish, etc.) As a result, AMNH:12345 could be a clam, fish, bird, reptile or mammal in New York City's natural history museum. Including the collectionCode in the literature reference identifies the specimen unambiguously.

## ﻿Linking Literature to Specimens﻿

**Figure 1.**
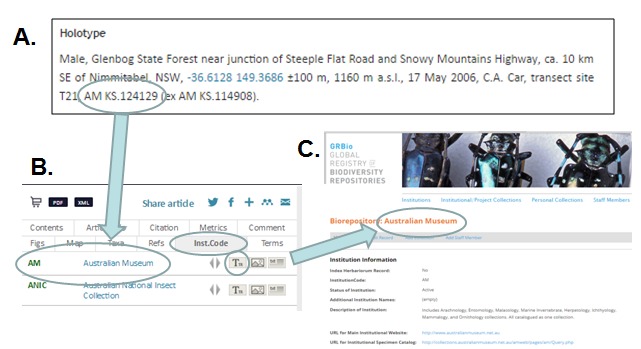
References to specimens in an article begin with an institutionCode that identifies the repository holding the specimen. A. A cited specimen includes a reference to an institution and catalog number (from Mesibov 2015p.143). B. The Inst.Code tab to the right of the ZooKeys article presents a list of all institutionCodes mentioned in the article. Each institutionCode listed on the Inst.Code tab is linked to the corresponding institution record in GRBio. The "T" icon next to each institution is linked to all mentions of that institution in the article (from index area, Mesibov 2015). C. The institution's name in B is hyperlinked to the corresponding GRBio record.

The publication of Mesibov (2015) made ZooKeys the first journal to use institutionCodes as a regulated, standardized data element to improve access to specimens referred to in articles. Specifically, this article was the first to use institutionCodes to link the specimens cited in the article to a record in GRBio for the repository in which each referenced specimens is preserved (see Fig. 1). (Weijola et al. 2016) provides a more extensive example of this feature.

## ﻿Benefits ﻿to the Community﻿

This use of GRBio as an authoritative reference resource helps to ensure that the institutionCodes included in published specimen identifiers are unambiguous, accurate, and provide information that will help researchers who want to locate and examine published specimens. Names and email addresses for collection managers are particularly important and for this reason they are required fields in GRBio.

GRBio also increases the visibility of institutions and their collections, whether or not their specimens have been digitized and are represented in online catalogs or aggregators such as VertNet or GBIF. It enables researchers to find undigitized collections with relevant study material using a variety of criteria and the advanced search function. GRBio is also a resource for analyzing the distribution and nature of institutions and collections and it provides useful information to collection managers for inter-institutional transactions.

One of the most far-reaching uses for GRBio is as a resolver of ambiguous codes and names for institutions and collections. The current locations of specimens cited in the literature have been made obscure by transfers among institutions, mergers, and changes in institutional names and codes. Citations of specimens in the literature can be outdated and uninterpretable, but GRBio can be used as a look-up table for finding codes and names that have been abandoned, as well as the newer codes and names that replaced them. This will be an important capability when mining specimen citations from the literature becomes commonplace.

## ﻿Conclusion﻿

Data standards such as the Darwin Core Standard have been critical in the advancement of biodiversity informatics. As part of the implementation of this standard, GRBio serves several important functions for the biological research community, such as providing linkages between outdated and current institutionCodes. Maintaining the completeness and currency of the data is labor-intensive and requires community participation. In particular, we call upon institution officials and collection managers to register their institutions and collections. Registering previously used institution names and institutionCodes in GRBio is important for tracing the whereabouts of specimens published under discontinued names and codes. We also seek input on ways to improve the database and the registry’s portal using the Contact GRBio link found at the bottom of each screen.
